# The role of FOLFIRINOX in metastatic pancreatic cancer: a meta-analysis

**DOI:** 10.1186/s12957-021-02291-6

**Published:** 2021-06-21

**Authors:** Beilei Zhang, Fengyan Zhou, Jiaze Hong, Derry Minyao Ng, Tong Yang, Xinyu Zhou, Jieyin Jin, Feifei Zhou, Ping Chen, Yunbao Xu

**Affiliations:** 1 The Second Clinical Medical College, Zhejiang Chinese Medical University, Hangzhou, Zhejiang China; 2 Emergency Medical Center, Ningbo Yinzhou No 2 Hospital, Ningbo, Zhejiang China; 3 Medical College of Ningbo University, Ningbo, Zhejiang China; 4 Department of Tumor HIFU Therapy, HwaMei Hospital, University of Chinese Academy of Sciences, Ningbo, Zhejiang China; 5 Department of General Surgery, HwaMei Hospital, University of Chinese Academy of Sciences, Ningbo, Zhejiang China; 6 Department of Radiotherapy and Chemotherapy, Hwamei Hospital, University of Chinese Academy of Sciences, Northwest Street 41, Haishu District, Ningbo, 315010 Zhejiang China

**Keywords:** Metastatic pancreatic cancer, FOLFIRINOX, Efficacy, Safety, Meta-analysis

## Abstract

**Background:**

The prognosis of pancreatic cancer (PC) is extremely poor, and most patients with metastatic PC still receive palliative care. Here, we report the efficacy and safety of FOLFIRINOX (oxaliplatin, irinotecan, leucovorin, 5-fluorouracil) in the treatment of metastatic PC.

**Methods:**

We searched PubMed, Web of Science, EBSCO, and Cochrane library databases for articles that described efficacy and safety of FOLFIRINOX in patients with metastatic PC, from January 1996 to July 2020. The primary outcomes targeted included overall survival (OS) and progression-free survival (PFS).

**Results:**

We found that FOLFIRINOX could directly improve OS rate of patients with metastatic PC (HR 0.76, 95% Cl 0.67–0.86, *p*<0.001) but had no benefit on PFS. Results from subgroup analyses showed that FOLFIRINOX had superior benefits than monochemotherapy (HR 0.59, 95% Cl 0.52–0.67, *p*<0.001), followed by FOLFIRINOX versus combination chemotherapy (HR 0.76, 95% Cl 0.61–0.95, *p*<0.001). The result of FOLFIRINOX versus nab-paclitaxel + gemcitabine had no benefit (HR 0.91, 95% Cl 0.82–1.02, *p*>0.05). The main adverse events (AEs) targeted hematological toxicity and the gastrointestinal system, and included febrile neutropenia, a reduction in white blood cells and appetite, as well as diarrhea.

**Conclusion:**

These findings indicated that FOLFIRINOX has potential benefits for the prognosis of patients with metastatic PC. Furthermore, there is no difference between the regimen of FOLFIRINOX and nab-paclitaxel + gemcitabine in this study. The application of FOLFIRINOX should be according to the actual situation of the patients and the experience of the doctors.

**Graphical abstract:**

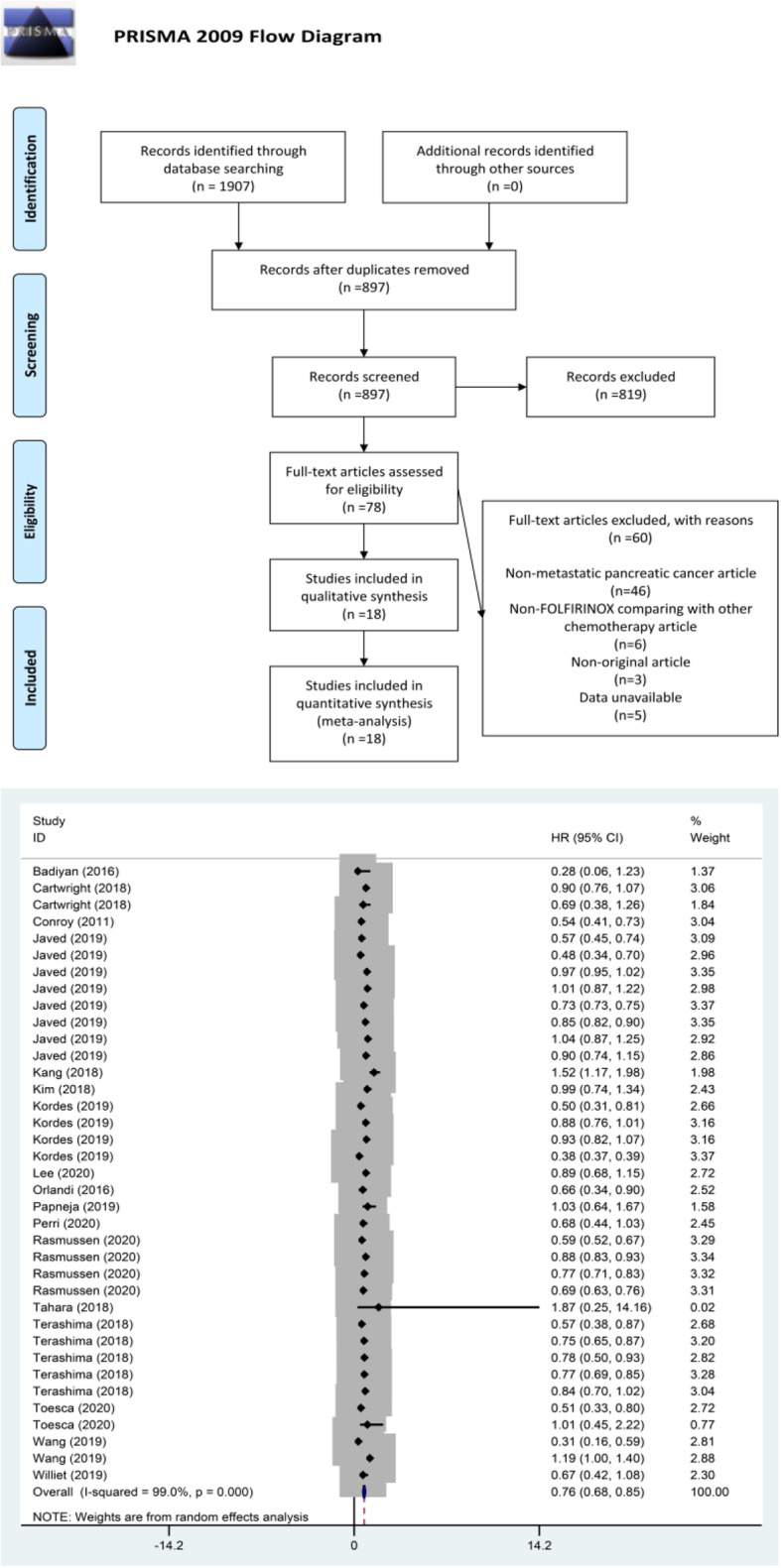

**Supplementary Information:**

The online version contains supplementary material available at 10.1186/s12957-021-02291-6.

## Introduction

The 2018 Global Cancer Incidence Research Report, based on 185 countries, regards pancreatic cancer (PC) as the seventh leading cause of cancer-related deaths worldwide [1]. PC has an extremely poor prognosis, with a 5-year relative survival rate of only 8% [2]. This is attributed to the fact that PC cases are either locally invasive or metastasized at diagnosis [3]. Despite recent progress in developing treatment therapies that improve PC outcomes, to date, approximately 20% of all patients survive at 1 year [4]. In fact, most approaches for treating metastatic PC still use palliative therapy [5]. A randomized trial by Burris et al. showed that gemcitabine resulted in a moderate survival advantage, compared to 5-FU treatment, as evidenced by median survival times of 5.65 and 4.41 months, respectively (*p*=0.0025) [6]. Consequently, gemcitabine has become a reference plan and the first-line drug for clinical treatment of metastatic PC [7]. On the other hand, previous studies have demonstrated the anti-tumor activity of irinotecan and oxaliplatin against several solid tumors, especially in gastrointestinal tumor cell lines [8]. In addition, a combination of leucovorin and 5-FU-based therapies applied for treatment of metastatic colorectal cancer showed improved efficacy and toxicity [9]. Conroy et al. conducted a randomized trial to explore the effectiveness of FOLFIRINOX (oxaliplatin, irinotecan, leucovorin, 5-fluorouracil) versus gemcitabine as a first-line chemotherapy regimen in patients with metastatic PC, and found that FOLFIRINOX exerted significant survival benefits (HR, 0.54; *p*<0.001) relative to gemcitabine, suggesting its potential as a first-line treatment for patients with metastatic PC [10]. We hypothesized that FOLFIRINOX may have toxicity-related problems, since it was combined with other chemotherapy drugs. Therefore, we conducted a meta-analysis on the efficacy and toxicity of FOLFIRINOX as a chemotherapy regimen, relative to other chemotherapies in patients with metastatic PC. Our findings are expected to reveal its benefits in patients with metastatic PC.

## Methods

### Literature search strategy

This meta-analysis was conducted according to the Preferred Reporting Item of the Systematic Review and Meta-Analysis Agreement (PRISMA-P) 2015 [11]. To identify relevant research articles describing the effect of FOLFIRINOX in unresectable PC, we systematically searched various electronic databases, including PubMed, Web of Science, EBSCO, and Cochrane Library, from January 1996 to July 2020. We used the following search terms to filter related articles: “FOLFOXIRI”, “mFOLFOXIRI”, “modified FOLFOXIRI”, “FOLFIRINOX”, “mFOLFIRINOX”, “modified FOLFIRINOX”, “irinotecan”, “oxaliplatin”, “leucovorin”, “5-fluorouracil”, “pancreatic cancer”, “pancreatic carcinoma”, “carcinoma of the pancreas”, “cancer of the pancreas”. Two researchers independently reviewed the articles’ abstracts, according to our selection criteria, then examined review articles and references of all retrieved articles to obtain other potentially relevant items. There is no additional registration information for this study.

### Inclusion and exclusion criteria

Articles that met the following criteria were included in the analysis: (1) studies were randomized controlled trials (RCTs) or observational studies; (2) all patients were diagnosed with unresectable PC; (3) patients in the experimental group received FOLFIRINOX or modified FOLFIRINOX regimens, whereas those in the control group received only monotherapy or other chemotherapy regimens; (4) survival outcomes, including OS and PFS, were extractable; and (5) in case of duplicate or constantly updated publications, the latest article was used in this study. Conversely, articles that met the following criteria were excluded: (1) those reporting non-original research; (2) research population comprising non-metastatic PC patients, such as postoperative adjuvant or neoadjuvant chemotherapy; (3) studies that did not compare FOLFIRINOX with other chemotherapy; (4) appropriate data could not be obtained; and (5) articles not written in English.

### Outcome measures

Retrieved articles reported different survival outcomes, including OS and PFS. Briefly, OS was defined as the length of time at the start of treatment to the date of death from any cause, whereas PFS referred to the length of time between initiation of therapy and objective tumor progression or death [12]. Hazard ratios (HR)/relative ratios (RR), including 95% confidence interval (CI), were used to assess the effect of FOLFIRINOX in metastatic PC. Data were directly extracted from the article or estimated according to the Kaplan-Meier survival curve.

### Data extraction and assessment of the risk of bias

Data from each study were independently extracted by two researchers, using a pre-designed data extraction table. In case of disagreements, a third researcher was invited to reach a majority opinion [13]. The recorded information included baseline characteristics such as author, year of publication, tumor type, study type, therapeutic regimen, and sample size, as well as survival outcomes including OS, PFS, and adverse events (AEs). We assessed the risks of bias in the RCTs using the Cochrane Collaboration Risk of Bias Assessment Tool, then evaluated methodologic quality of observational studies using the Newcastle–Ottawa Quality Assessment Scale [14].

### Statistical analysis

All data analyses were performed in the Stata 12.0 software (Stata, College Station). Briefly, heterogeneity among studies was evaluated using the Cochran chi-square test [15] and heterogeneity statistic (I^2^) [16], whereas HR estimates were weighted and pooled using the Mantel-Haenszel random effects model to increase credibility by the regimen and the diversity of the population. In addition, we evaluated publication bias using Egger’s test [17, 18] and performed sensitivity analysis to assess stability of the results. Data followed by *p*<0.05 were considered statistically significant.

## Results

### Eligible research and inclusion characteristics

**Fig. 1:**
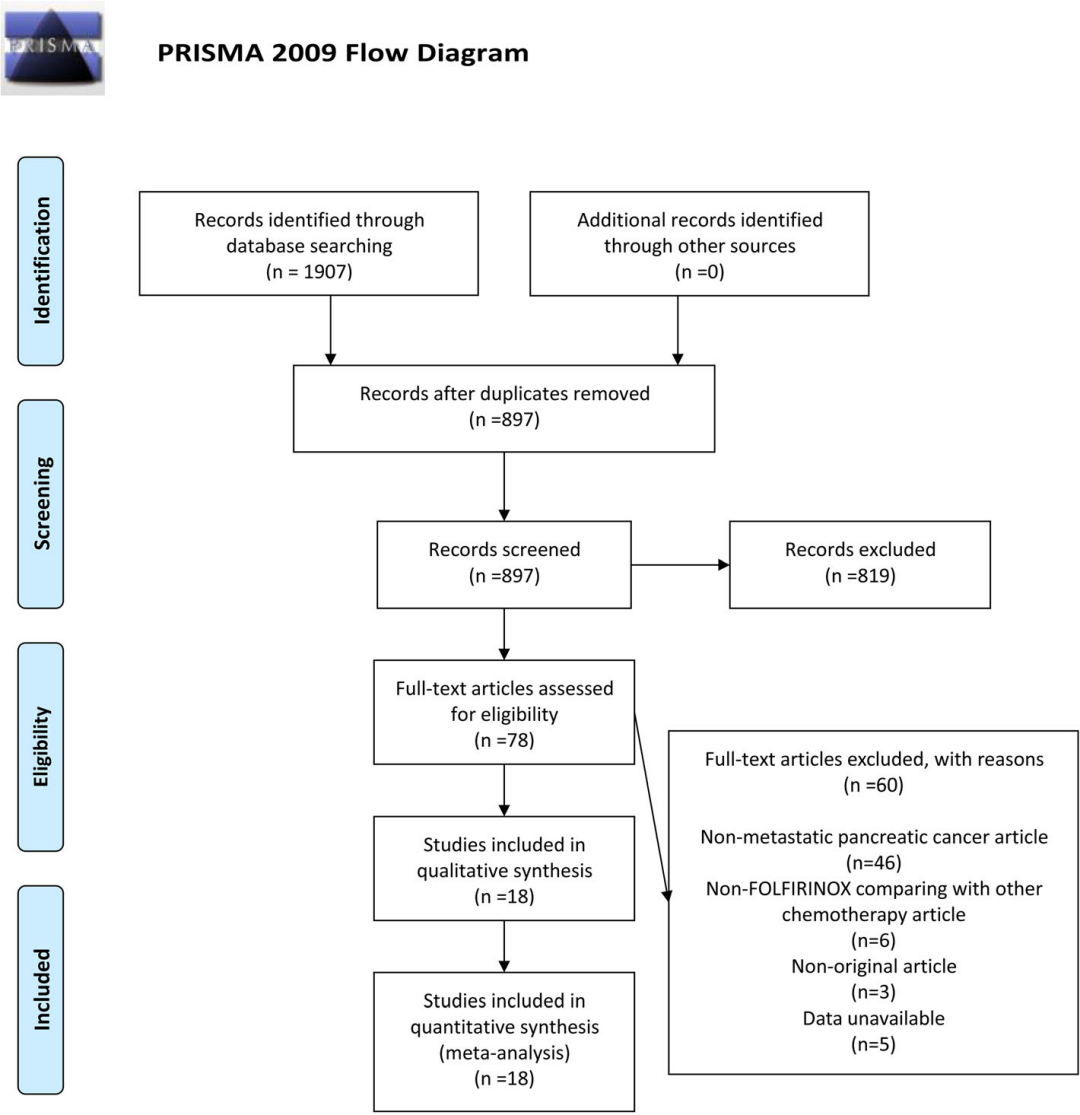
Flow diagram describing inclusion and exclusion criteria

Our search strategy resulted in a total of 1907 studies on FOLFIRINOX. After excluding non-metastatic PC, non-FOLFIRINOX compared with other chemotherapy, non-original, as well as studies whose data could not be extracted, a total of 18 articles [5, 10, 19–34] were finally included in our analysis. A detailed outline of the search and selection process is shown in Fig. 1. The included articles comprised RCT and retrospective studies, respectively, one and seventeen [5, 10, 19–34].

Eight studies [5, 10, 20, 23, 26, 29, 31, 33] described use of FOLFIRINOX versus monochemotherapy; most of which were FOLFIRINOX versus gemcitabine alone, 15 articles [5, 20–25, 27–34] related to FOLFIRINOX versus nab-paclitaxel combined with gemcitabine, whereas the rest reported FOLFIRINOX versus other chemotherapy regimens. The studies analyzed a total of 7556 participants, 2435 of whom received FOLFIRINOX. Among them, Kordes et al.’s research experimental group was FOLFOXIRI (oxaliplatin, irinotecan, 5-fluorouracil) [23]. Detailed characteristics of the patients are shown in Supplementary Table 1. We also assessed the quality of all incorporated retrospective as well as RCT studies using the Newcastle-Ottawa Quality Assessment Scale and the Cochrane Collaboration’s tool, respectively. Both sets of results showed reliable article quality (Supplementary Table 2).

### Effect of FOLFIRINOX on overall survival

**Fig. 2:**
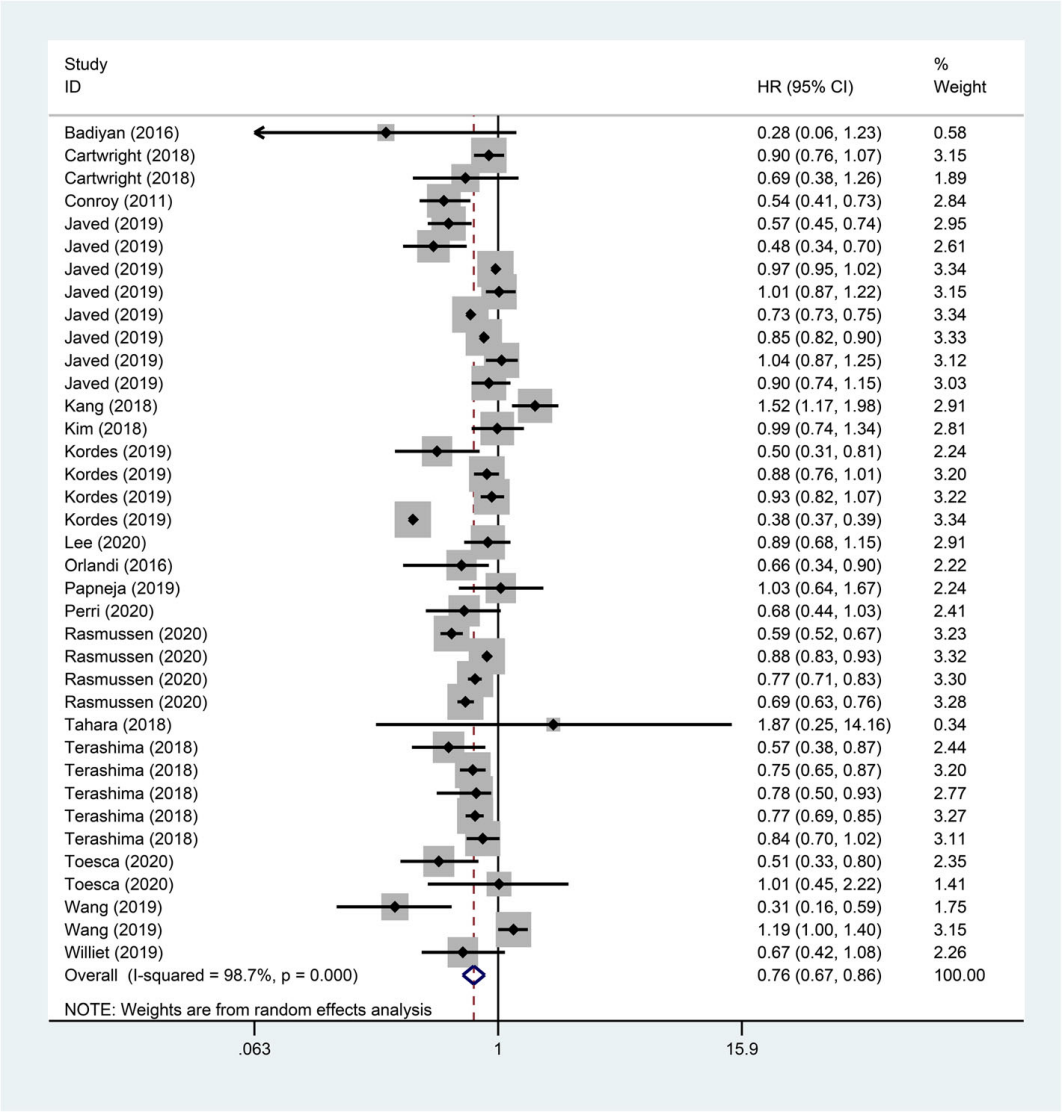
Forest plots of the overall survival for FOLFIRINOX on metastatic pancreatic cancer

**Fig. 3:**
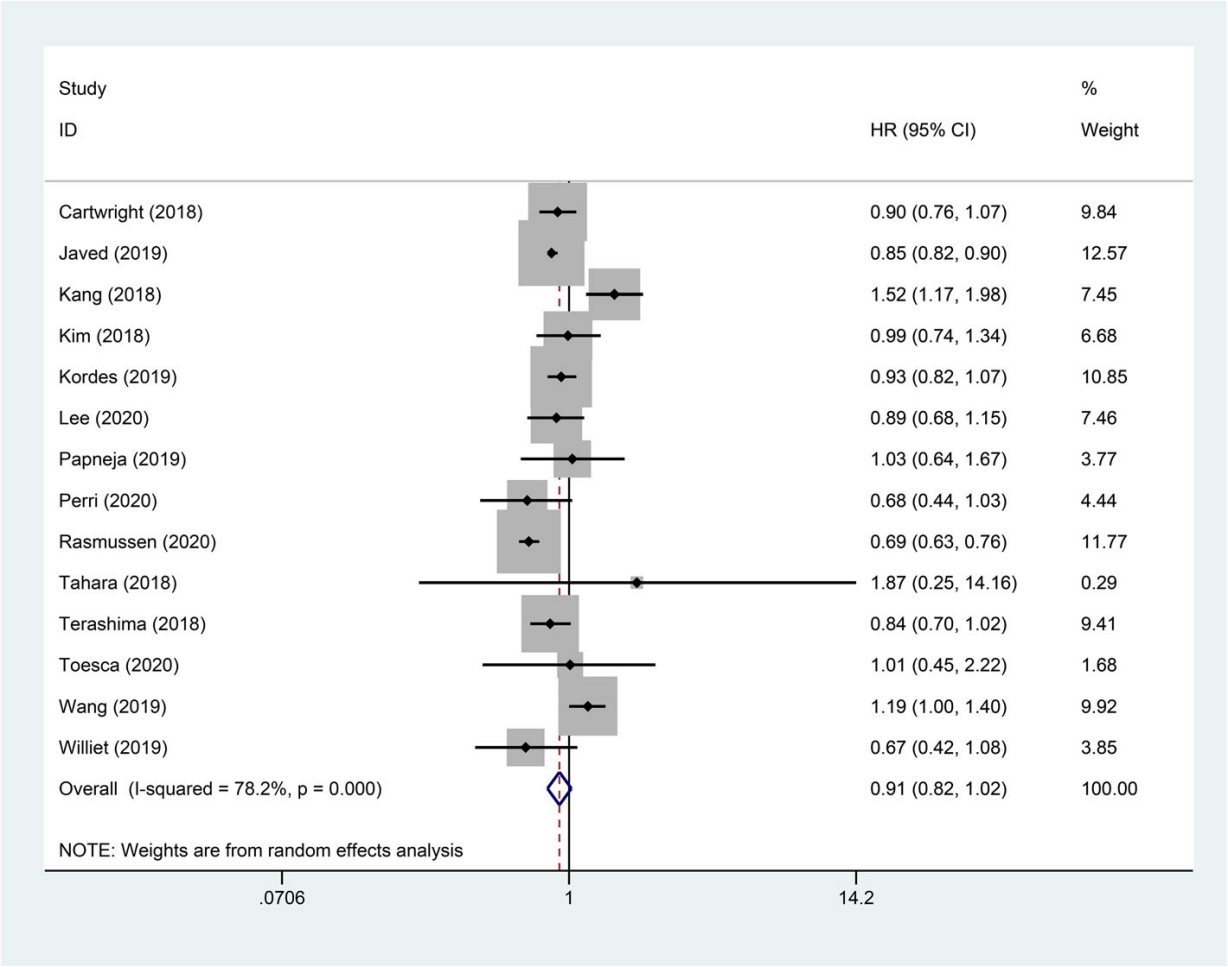
Forest plot of the overall survival for FOLFIRINOX versus nab-paclitaxel+gemcitabine on metastatic pancreatic cancer

In the study cohort, 17 studies [5, 10, 19–24, 26–34] reported OS with a combined total effect rate (HR 0.76, 95% Cl 0.67–0.86, *p*<0.001; Fig. 2). Due to the considerable heterogeneity of the results, we performed a subgroup analysis on OS. Although we conducted a subgroup analysis, the high heterogeneity was still inevitable. This was due to the diversity of the population and regimen we included, and it is precisely because of this that we used the random effects model in the selection of the model. It found that FOLFIRINOX had superior benefits compared with monochemotherapy, with a combined total effective rate of HR 0.59, 95% Cl 0.52–0.67, *p*<0.001 (Supplementary Fig. 1). This was followed by FOLFIRINOX versus combination chemotherapy, with a combined total effective rate of HR 0.76, 95% Cl 0.61–0.95, *p*<0.001 (Supplementary Fig. 2). The result of FOLFIRINOX versus nab-paclitaxel + gemcitabine had no benefit (HR 0.91, 95% Cl 0.82–1.02, *p*>0.05; Fig. 3).

### Effect of FOLFIRINOX on progression-free survival

We found eight studies [10, 21, 24–26, 32–34] that used PFS as endpoints, and subsequently combined them using statistical methods. The results revealed no benefit to PFS in the experimental group using FOLFIRINOX, relative to the control group (HR 0.88, 95% Cl 0.63–1.22, *p*>0.05; Supplementary Fig. 3).

### AEs of FOLFIRINOX

Among the included articles, a total of 12 studies [5, 10, 21–25, 27–31] described AEs. Analysis showed that total AEs were all hematological toxicity and gastrointestinal events, and included febrile neutropenia (RR=2.19), decreased white blood cell (RR=1.54), and low appetite (RR=1.58), as well as diarrhea (RR=2.72), mucositis (RR=3.84), nausea (RR=1.94), and vomiting (RR=1.63). Significant AEs, among those rated grade 3 and higher, included febrile neutropenia (RR=2.84), neutropenia (RR=1.67), white blood cell decreased (RR=3.09), diarrhea (RR=3.74), elevated ALT (RR=0.38), nausea (RR=3.60), and vomiting (RR=1.46) (Table 1).

**Table 1:** Subgroup analysis of the adverse events (AEs)

FOLFIRINOX vs. control	No. of studies	RR	95%CI	*p*	Heterogeneity (I^2^)
Any grade about neuropathy	6	1.12	0.60–2.10	0.72	83
Any grade anemia	8	0.95	0.59–1.52	0.84	94
Any grade decreased appetite	3	1.58	1.08–2.30	*0.02*	78
Any grade diarrhea	6	2.72	1.60–4.61	*<0.001*	74
Any grade fatigue	5	1.25	0.92-1.71	0.16	70
Any grade febrile neutropenia	4	2.19	1.04–4.65	*0.04*	25
Any grade mucositis	2	3.84	2.33–6.34	*<0.001*	0
Any grade nausea	4	1.94	1.16–3.24	*0.01*	46
Any grade neutropenia	8	1.15	0.86–1.52	0.35	90
Any grade thrombocytopenia	7	0.76	0.42–1.36	0.36	92
Any grade vomiting	4	1.63	1.26–2.09	*<0.001*	0
Any grade white blood cell decrease	2	1.54	1.16–2.04	*0.003*	6
Grade 3 or higher about neuropathy	7	0.85	0.33–2.18	0.73	62
Grade 3 or higher anemia	9	1.21	0.73–2.03	0.46	31
Grade 3 or higher decreased appetite	2	1.41	0.56–3.57	0.47	0
Grade 3 or higher diarrhea	8	3.74	1.62–8.62	*0.002*	54
Grade 3 or higher elevated ALT	3	0.38	0.21–0.68	*0.001*	0
Grade 3 or higher fatigue	6	0.95	0.62–1.45	0.80	35
Grade 3 or higher febrile neutropenia	6	2.84	1.35–5.99	*0.006*	30
Grade 3 or higher mucositis	2	1.34	0.71–2.51	0.37	NA
Grade 3 or higher nausea	4	3.60	1.26–10.30	*0.02*	11
Grade 3 or higher neutropenia	8	1.67	1.14–2.46	*0.009*	87
Grade 3 or higher thrombocytopenia	9	1.20	0.74–1.93	0.47	18
Grade 3 or higher vomiting	6	1.46	1.07–1.98	*0.02*	0
Grade 3 or higher white blood cell decrease	2	3.09	2.00–4.77	*<0.001*	0
Death	2	0.49	0.20–1.23	0.13	0

*RR* risk ratio, *ALT* alanine aminotransferase, *NA* not applicable

Data in italics are statistically siginificant (P<0.05)

### Publication bias and sensitivity of the OS of FOLFIRINOX

Due to the relatively large number of articles included in our analysis, we evaluated publication bias to ascertain the relationship between OS and FOLFIRINOX. Egger *p*=0.446, showing there was no publication bias. Results from the sensitivity analysis showed stability.

## Discussion

Our results showed that FOLFIRINOX was the most potentially beneficial regimen in metastatic PC, but in terms of long-time benefits, there was no statistical difference compared with nab-paclitaxel + gemcitabine. Interestingly, Kang et al. [21] and Papneja et al. [27] also reported that FOLFIRINOX had no significant benefit versus nab-paclitaxel + gemcitabine in patients with metastatic PC. However, Suker et al. [35] found that FOLFIRINOX could prolong the overall survival of patients with locally advanced PC by comparison of FOLFIRINOX and gemcitabine. Interestingly, two studies of neoadjuvant chemotherapy based on FOLFIRINOX have also been confirmed to benefit patients with locally advanced PC [36, 37]. Furthermore, FOLFIRINOX and nab-paclitaxel + gemcitabine had superior benefits to the other group with regards to survival of patients with metastatic PC, consistent with other recent studies that reported similar findings [20, 23, 29].

From an OS perspective, the use of total FOLFIRINOX can benefit patients with metastatic PC, owing to a direct increase in the OS rate of disease control. However, results from our heterogeneity analysis were relatively large, necessitating further validation. In addition, results from subgroup analysis of the heterogeneity indicated that FOLFIRINOX exerted survival benefits irrespective of whether the control group comprised monotherapy or gemcitabine-based combination chemotherapy, consistent with Orlandi et al. [26] and Terashima et al. [31] who found significantly higher survival benefits in the FOLFIRINOX relative to the control group. Similarly, results from a retrospective study on unresectable PC [32] also showed that FOLFIRINOX may have a survival benefit. Conversely, reported contrasting results [30]. We hypothesized that the conclusion of Tahara et al. might be attributed to the small sample size used in their study. Because the control group of this article contained different chemotherapy regimens, from the perspective of the first-line chemotherapy regimen of nab-paclitaxel + gemcitabine as the control group, our results proved that the regimen of FOLFIRINOX has potential survival benefit trend on patients with metastatic PC, consistent with previous studies [5, 28].

FOLFIRINOX had superior OS benefits relative to monotherapy, followed by FOLFIRINOX versus gemcitabine-based combination chemotherapy not included nab-paclitaxel + gemcitabine. In the monotherapy and combination chemotherapy regimen, gemcitabine accounted for the majority of the benefit. Therefore, we attributed this difference in efficacy to the mechanism of drug action. As a deoxycytidine analog of gemcitabine, its cytotoxic activity was based on several activities of DNA synthesis. Functionally, structural differences between the fluorine substituents on the 2’position of the furanose ring of gemcitabine gives gemcitabine the unique cellular pharmacological characteristics, metabolism, and mechanism action with other nucleoside analogs [38]. However, gemcitabine is highly resistant, which appears within a few weeks of chemotherapy [39]. The mechanism may be caused by an alteration of gemcitabine drug metabolism that causes incorporation of cytidine analogs into DNA [40], or it may be related to a reduction of gemcitabine-induced apoptosis [41, 42].

Results from our subgroup analysis of FOLFIRINOX versus nab-paclitaxel + gemcitabine showed that the overall trend was biased towards the regimen of FOLFIRINOX; however, there was no statistical difference, which may be related to the small sample size. With the expansion of the sample size, the benefit trend of FOLFIRINOX may appear. For the combination chemotherapy of nab-paclitaxel + gemcitabine, its benefits were crucial in clinical treatment. Related studies have shown that the combination of nab-paclitaxel + gemcitabine can increase the drug concentration of gemcitabine in tumor cells, thereby causing cytotoxicity [43]. And this combination medication program was widely used in clinical practice. For the regimen of FOLFIRINOX, given the relatively large toxicity of FOLFIRINOX reported in previous studies and the higher physical requirements of this program [44], the population’s clinical application is not extensive and needs careful consideration. Furthermore, PFS had no benefit, possibly due to the small sample size and insufficient follow-up time [25, 26]. This may also be attributed to the difference between indicators setting up in each article and the type of study (whether it is RCT). Despite FOLFIRINOX’s superior benefits compared to the control regimen, further research is needed to evaluate its clinical value.

In the FOLFIRINOX program, previous studies have shown that irinotecan exerts specific clinical activity in patients with metastatic PC [45, 46]. Similarly, preclinical studies by Azrak et al. [47], Mans et al. [48], and Mullany et al. [49] have shown that a combination of irinotecan with calcium folinate and fluorouracil produces significant synergistic effect [50]. Interestingly, clinical trials by Ducreux et al. [51] revealed that oxaliplatin produces individual clinical activity against PC only when combined with fluorouracil. Furthermore, a synergistic effect is produced when combined with irinotecan in vitro [52]. Consequently, Ychou et al. [8] and Conroy et al. [53] explored the benefits of irinotecan, oxaliplatin, fluorouracil, and calcium leucovorin in patients with metastatic PC and found that FOLFIRINOX had encouraging clinical benefits.

Researchers need to consider both the efficacy and toxicity before extensive clinical applications. Therefore, anticancer drugs’ AEs are a vital consideration in clinical oncology [54]. AEs associated with FOLFIRINOX mainly targeted the blood and gastrointestinal systems. We speculated that this might be positively correlated with the mechanism of action of the drug itself. Previous studies have shown that, as a third-generation platinum compound [55], bone marrow suppression is a joint AE of oxaliplatin, and can cause blood system toxicity such as thrombocytopenia. Other studies have shown that the blood system toxicity of oxaliplatin may also be related to the immune-dependent mechanism [56] and the induced spleen enlargement [57]. Regarding the gastrointestinal system, we speculated that AE occurrence might be related to the anatomical location of the tumor.

FOLFIRINOX toxicity is a problem that cannot be ignored. This is because it had clear benefits, but was also accompanied by relatively large toxicities [58]. Consequently, mFOLFIRINOX has been employed as a potential agent for reducing toxicity and achieved significant curative effects. For instance, Kang et al. [59] and Ghorani et al. [60] reported that mFOLFIRINOX generated comparable efficacies to FOLFIRINOX in patients with metastatic PC, and this was accompanied by weak toxicity. They suggested that if clinically necessary, 75% of the standard dose should be used for treatment, and can alleviate the toxicity without affecting the efficacy. Unfortunately, this study did not include more related clinical trials on mFOLFIRINOX. Therefore, regarding the problem of FOLFIRINOX toxicity, we have put forward the following suggestions: (1) in the choice of clinical regimen, we recommend using 75% of the standard dose to treat patients with metastatic PC; (2) we recommend regular monitoring of the patient’s various indicators to understand the patient’s physical function during the treatment process; (3) in the selection of patients with metastatic PC, we recommend more patients with higher physical fitness scores to receive the treatment of this program in order to achieve better curative effects [61]. All in all, there are more clinical trials needed to evaluate ways of minimizing FOLFIRINOX toxicity to enhance its use in the clinical treatment of metastatic PC.

### Limitations

Our analysis included several retrospective studies, which could have compromised accuracy of our results. Even though a few non-metastatic PC cases are contained in the included 12 articles, we have no way to obtain data for subgroup analysis. Thus, the subject of our study is metastatic PC, and we are discussing according to the treatments. In addition, the diverse treatment options in the control group may have generated potential heterogeneity, although we comprehensively and systematically compared efficacy of FOLFIRINOX as the experimental group in patients with metastatic PC. Nevertheless, the different medication regimens used indicated that FOLFIRINOX had more apparent benefits affirming our conclusions. However, these conclusions need to be validated using a large number of clinical trials.

### Strengths

Although this article has the above limitations, it can still provide further strong evidence for the chemotherapy of metastatic PC, and it is hopeful to be widely used in clinical practice for the treatment of metastatic PC.

## Conclusion

FOLFIRINOX is the potentially optimal regimen for the prognosis of patients with metastatic PC. Although the AEs increased at different degrees, FOLFIRINOX is generally safe and tolerable. There is no difference between the regimen of FOLFIRINOX and nab-paclitaxel + gemcitabine in this study. Therefore, it remains debatable whether the regimen of FOLFIRINOX can replace the combination chemotherapy of nab-paclitaxel + gemcitabine and the selection of the specific implementation plan should be according to the actual situation of the patients and the experience of the doctors.

## Supplementary Information

**Additional file 1: Supplementary Fig. 1.** Forest plot of the overall survival for FOLFIRINOX versus mono-chemotherapy on metastatic pancreatic cancer.

**Additional file 2: Supplementary Fig. 2.** Forest plot of the overall survival for FOLFIRINOX versus combination chemotherapy on metastatic pancreatic cancer.

**Additional file 3: Supplementary Fig. 3.** Forest plots of the progression-free survival for FOLFIRINOX on metastatic pancreatic cancer.

**Additional file 4: Supplementary Table 1.** Characteristics of included clinical trials in the meta-analysis.

**Additional file 5: Supplementary Table 2.** Quality assessment of studies included.

## Data Availability

The datasets supporting the conclusions of this article are included within the article.

## Abbreviations

PC: Pancreatic cancer
FOLFIRINOX: Oxaliplatin, irinotecan, leucovorin, 5-fluorouracil
FOLFOXIRI: Oxaliplatin, irinotecan, 5-fluorouracil
OS: Overall survival
PFS: Progression-free survival
AEs: Adverse events
HR: Hazard ratios
RR: Relative risk
CI: Confidence interval
RCT: Randomized controlled trial
