# Supplementary figures and images for: The role of FOLFIRINOX in metastatic pancreatic cancer: a meta-analysis
Source: World J Surg Oncol. 2021 Jun 21;19:182. doi: 10.1186/s12957-021-02291-6 (PMC8218408; doi:10.1186/s12957-021-02291-6)

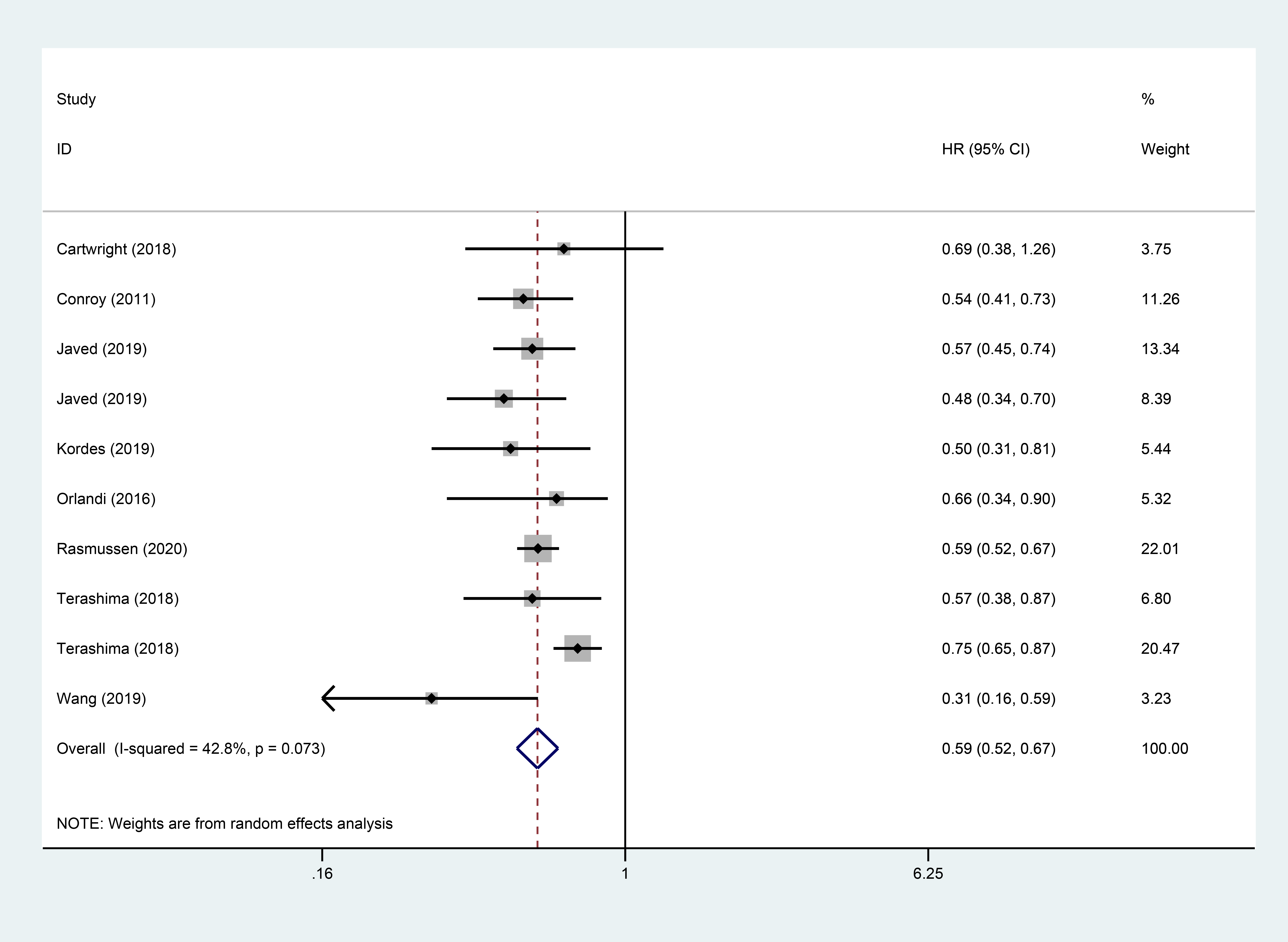

Supplement: Supplementary file 1 — Additional file 1: Supplementary Fig. 1. Forest plot of the overall survival for FOLFIRINOX versus mono-chemotherapy on metastatic pancreatic cancer. [file 12957_2021_2291_MOESM1_ESM.tif]

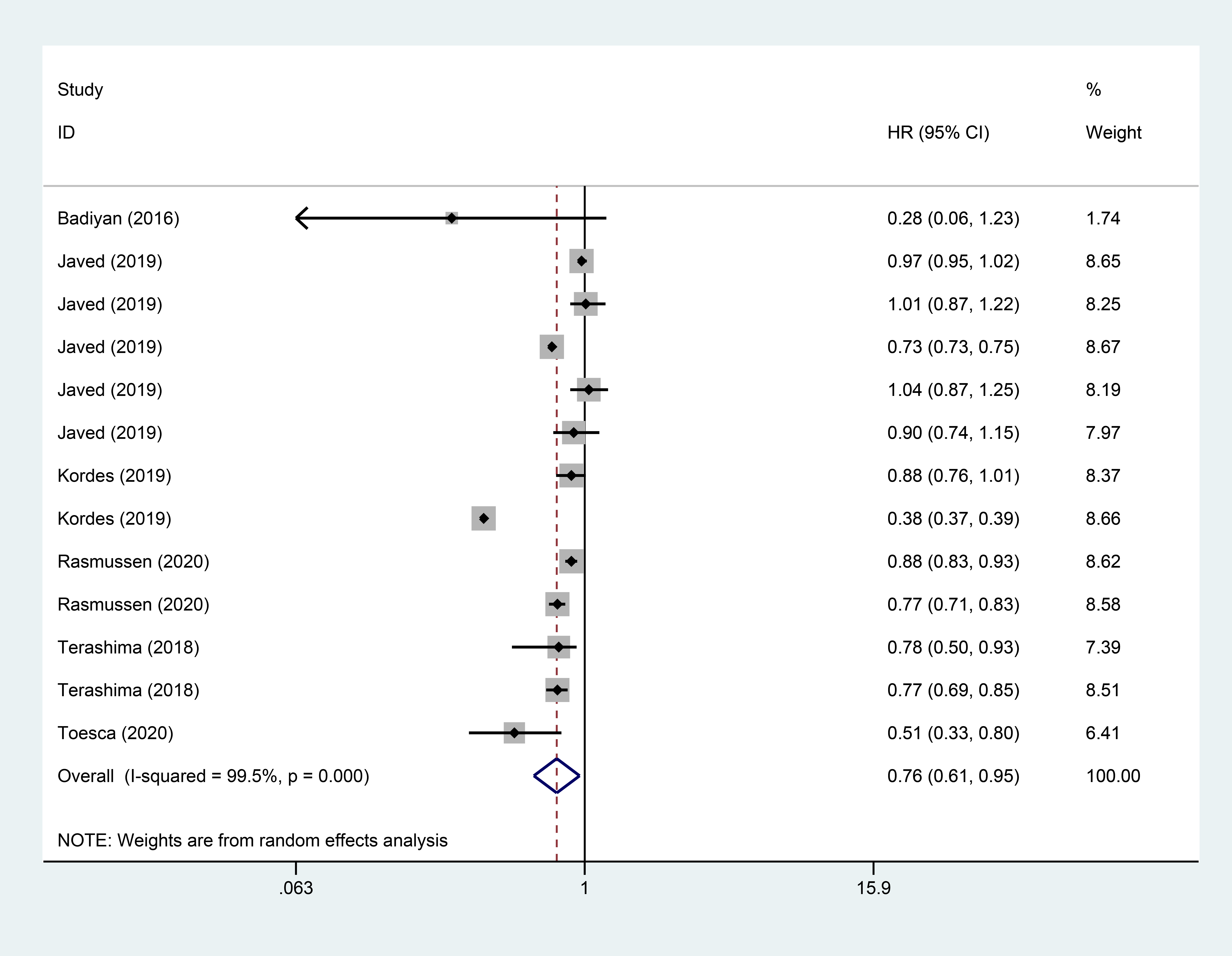

Supplement: Supplementary file 2 — Additional file 2: Supplementary Fig. 2. Forest plot of the overall survival for FOLFIRINOX versus combination chemotherapy on metastatic pancreatic cancer. [file 12957_2021_2291_MOESM2_ESM.tif]

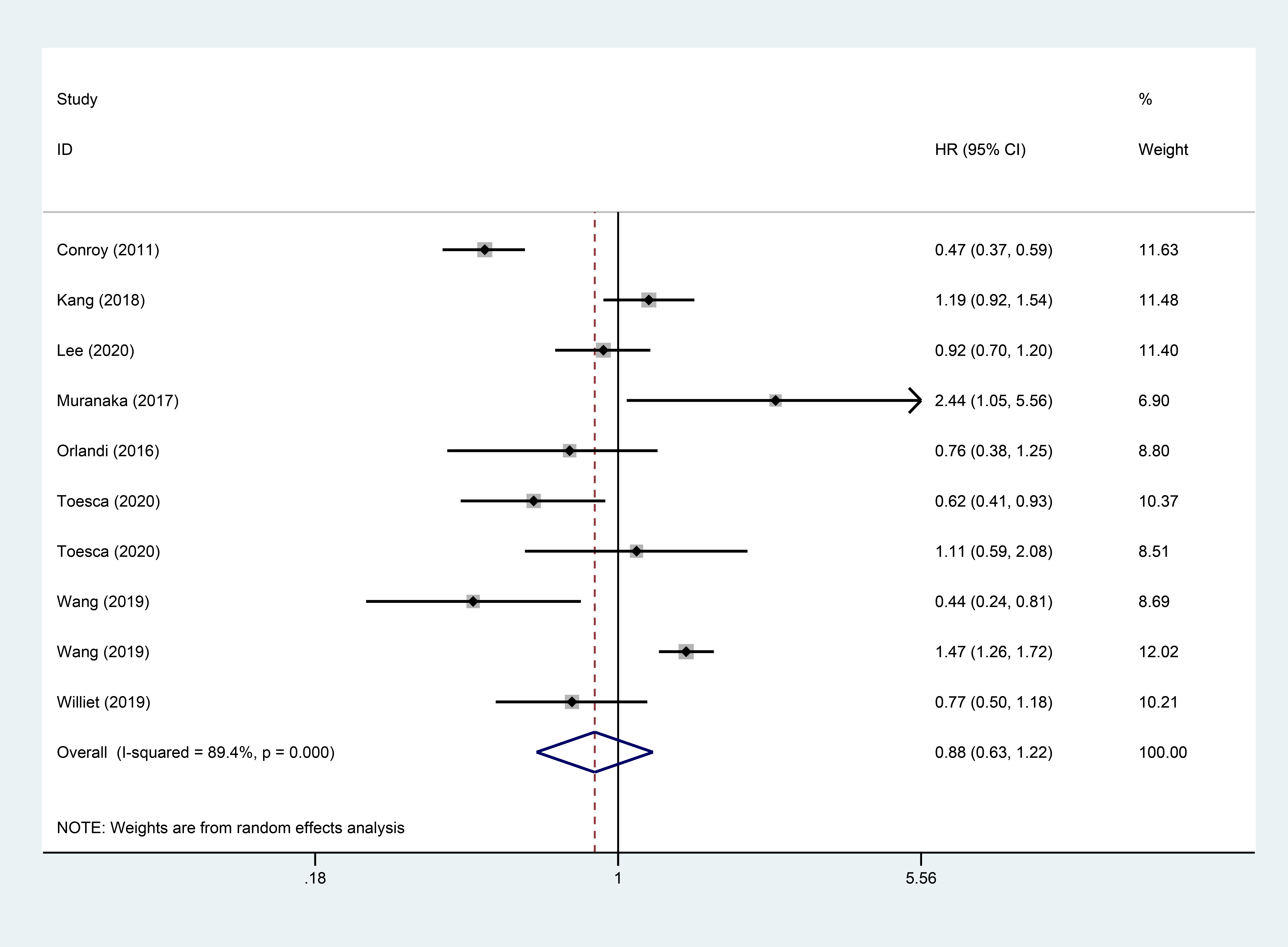

Supplement: Supplementary file 3 — Additional file 3: Supplementary Fig. 3. Forest plots of the progression-free survival for FOLFIRINOX on metastatic pancreatic cancer. [file 12957_2021_2291_MOESM3_ESM.tif]
