# Supplementary material for: The role of FOLFIRINOX in metastatic pancreatic cancer: a meta-analysis
Source: World J Surg Oncol. 2021 Jun 21;19:182. doi: 10.1186/s12957-021-02291-6 (PMC8218408; doi:10.1186/s12957-021-02291-6)
Supplement: Supplementary file 4 — Additional file 4: Supplementary Table 1. Characteristics of included clinical trials in the meta-analysis. [file 12957_2021_2291_MOESM4_ESM.docx]

**Supplementary Table 1**. Characteristics of included clinical trials in the meta-analysis.

| Author, year | Tumor type | Study Type | Therapeutic regimen | | Number of inclusions | |
| --- | --- | --- | --- | --- | --- | --- |
|  |  |  | Treatment | Control | Treatment | Control |
| Badiyan, 2016 | Borderline Resectable and Locally Advanced PC | Retrospective Study | FOLFIRINOX | Gemcitabine-based therapy | 15 | 17 |
| Cartwright, 2018 | Metastatic PC | Retrospective Study | FOLFIRINOX | Nab-paclitaxel + Gemcitabine, Gemcitabine | 159 | 327 |
| Conroy, 2011 | Metastatic PC | RCT | FOLFIRINOX | Gemcitabine | 171 | 171 |
| Javed, 2019 | Metastatic PC | Retrospective Study | FOLFIRINOX | Gemcitabine, Nab-paclitaxel + Gemcitabine, Other gemcitabine or 5-FU-based regimens | 204 | 826 |
| Kang, 2018 | Metastatic PC | Retrospective Study | FOLFIRINOX | Nab-paclitaxel + Gemcitabine | 159 | 149 |
| Kim, 2018 | Metastatic PC | Retrospective Study | FOLFIRINOX | Nab-paclitaxel + Gemcitabine | 317 | 337 |
| Kordes, 2019 | Advanced PC | Retrospective Study | 5-FU+oxaliplatin+irinotecan | Gemcitabine, Gemcitabine + Capecitabine, Gemcitabine + nab-paclitaxel, 5-FU + oxaliplatin | 31 | 564 |
| Lee, 2020 | Metastatic PC | Retrospective Study | FOLFIRINOX | Nab-paclitaxel + Gemcitabine | 232 | 181 |
| Muranaka, 2017 | Unresectable PC | Retrospective Study | FOLFIRINOX | Nab-paclitaxel + Gemcitabine | 16 | 22 |
| Orlandi, 2016 | Advanced PC | Retrospective Study | FOLFIRINOX | Gemcitabine | 36 | 34 |
| Papneja, 2019 | Advanced PC | Retrospective Study | FOLFIRINOX | Nab-paclitaxel + Gemcitabine | 86 | 33 |
| Perri, 2020 | Localized PC | Retrospective Study | FOLFIRINOX | Nab-paclitaxel + Gemcitabine | 285 | 200 |
| Rasmussen, 2020 | PC | Retrospective Study | FOLFIRINOX | Gemcitabine-based chemotherapy | 435 | 1280 |
| Tahara, 2018 | Advanced PC | Retrospective Study | FOLFIRINOX | Nab-paclitaxel + Gemcitabine | 12 | 15 |
| Terashima, 2018 | Unresectable PC | Retrospective Study | FOLFIRINOX | Chemotherapy | 47 | 616 |
| Toesca, 2020 | Unresectable PC | Retrospective Study | modified FOLFIRINOX | Gemcitabine-based chemotherapy | 31 | 107 |
| Wang, 2019 | Advanced PC | Retrospective Study | FOLFIRINOX | Nab-paclitaxel + Gemcitabine, Gemcitabine | 92 | 133 |
| Williet, 2019 | Metastatic PC | Retrospective Study | FOLFIRINOX | Nab-paclitaxel + Gemcitabine | 107 | 109 |

PC: pancreatic cancer; RCT: randomized controlled trial; FOLFIRINOX: irinotecan, oxaliplatin, leucovorin, 5-fluorouracil; modified FOLFIRINOX: modified combination of 5-fluorouracil, leucovorin, irinotecan, oxaliplatin; Nab-paclitaxel, nanoparticle albumin-bound paclitaxel; 5-FU: 5-fluorouracil.
