# Supplementary material for: The role of FOLFIRINOX in metastatic pancreatic cancer: a meta-analysis
Source: World J Surg Oncol. 2021 Jun 21;19:182. doi: 10.1186/s12957-021-02291-6 (PMC8218408; doi:10.1186/s12957-021-02291-6)
Supplement: Supplementary file 5 — Additional file 5: Supplementary Table 2. Quality assessment of studies included. [file 12957_2021_2291_MOESM5_ESM.docx]

**Supplementary Table 2.** Quality assessment of studies included.

| Author, year,  Study (RCT) | Sequence  Generation | | Allocation  Concealment | | Blinding | Incomplete  outcome data | | Selective  outcome reporting | | Free of  other bias |  |  |  |  |  |
| --- | --- | --- | --- | --- | --- | --- | --- | --- | --- | --- | --- | --- | --- | --- | --- |
| Conroy, 2011 | low risk | | low risk | | unclear risk | low risk | | low risk | | low risk |  |  |  |  |  |
| Author, year,  Study (Observational) | | **Selection (Out of 4)** | | | | | | | | | **Comparability**  **(Out of 2)** | **Outcomes(Out of 3)** | | | **Total**  **(Out of 9)** |
|  |  | Representativeness of exposed cohort | | Selection of nonexposed cohort | | | Ascertainment  of exposure | | Outcome not present at the start of the study | |  | Assessment of outcomes | Length of follow-up | Adequacy of follow-up |  |
| Badiyan, 2016 | | 1 | | 0 | | | 1 | | 1 | | 1 | 1 | 1 | 1 | 7 |
| Cartwright, 2018 | | 1 | | 0 | | | 1 | | 1 | | 2 | 1 | 1 | 1 | 8 |
| Javed, 2019 | | 1 | | 1 | | | 1 | | 1 | | 2 | 1 | 1 | 0 | 8 |
| Kang, 2018 | | 1 | | 0 | | | 1 | | 1 | | 2 | 1 | 1 | 0 | 7 |
| Kim, 2018 | | 1 | | 1 | | | 1 | | 1 | | 1 | 1 | 1 | 1 | 8 |
| Kordes, 2019 | | 1 | | 0 | | | 1 | | 1 | | 2 | 1 | 1 | 1 | 8 |
| Lee, 2020 | | 1 | | 1 | | | 1 | | 1 | | 2 | 1 | 0 | 0 | 7 |
| Muranaka, 2017 | | 1 | | 0 | | | 1 | | 1 | | 1 | 1 | 1 | 1 | 7 |
| Orlandi, 2016 | | 1 | | 1 | | | 1 | | 1 | | 1 | 1 | 0 | 0 | 6 |
| Papneja, 2019 | | 1 | | 0 | | | 1 | | 1 | | 2 | 1 | 1 | 0 | 7 |
| Perri, 2020 | | 1 | | 1 | | | 1 | | 1 | | 2 | 1 | 1 | 0 | 8 |
| Rasmussen, 2020 | | 1 | | 1 | | | 1 | | 1 | | 2 | 1 | 0 | 0 | 7 |
| Tahara, 2018 | | 1 | | 1 | | | 1 | | 1 | | 1 | 1 | 0 | 0 | 6 |
| Terashima, 2018 | | 1 | | 0 | | | 1 | | 1 | | 2 | 1 | 1 | 1 | 8 |
| Toesca, 2020 | | 1 | | 0 | | | 1 | | 1 | | 2 | 1 | 1 | 0 | 7 |
| Wang, 2019 | | 1 | | 0 | | | 1 | | 1 | | 2 | 1 | 1 | 1 | 8 |
| Williet, 2019 | | 1 | | 1 | | | 1 | | 1 | | 2 | 1 | 1 | 0 | 8 |

The RCTs and observational studies were assessed by the Cochrane Collaboration’s tool and Newcastle-Ottawa Quality Assessment Scale, respectively.

Risk of bias was assessed as “low risk”, “high risk” or “unclear risk”.
